# Biochemical and functional characterization of *Plasmodium falciparum* GTP cyclohydrolase I

**DOI:** 10.1186/1475-2875-13-150

**Published:** 2014-04-19

**Authors:** Krittikorn Kümpornsin, Namfon Kotanan, Pornpimol Chobson, Theerarat Kochakarn, Piyaporn Jirawatcharadech, Peera Jaru-ampornpan, Yongyuth Yuthavong, Thanat Chookajorn

**Affiliations:** 1 Department of Biochemistry, Faculty of Science, Mahidol University, Bangkok 10400, Thailand; 2 National Center for Genetic Engineering and Biotechnology, National Science and Technology Development Agency, Pathum Thani 12120, Thailand; 3 Center of Excellence in Malaria, Faculty of Tropical Medicine, Mahidol University, Bangkok 10400, Thailand; 4 Mahidol Vivax Research Unit, Center of Excellence in Malaria, Faculty of Tropical Medicine, Mahidol University, Bangkok 10400, Thailand

**Keywords:** Antifolate, Folate pathway, GTP cyclohydrolase I, Malaria

## Abstract

**Background:**

Antifolates are currently in clinical use for malaria preventive therapy and treatment. The drugs kill the parasites by targeting the enzymes in the *de novo* folate pathway. The use of antifolates has now been limited by the spread of drug-resistant mutations. GTP cyclohydrolase I (GCH1) is the first and the rate-limiting enzyme in the folate pathway. The amplification of the *gch1* gene found in certain *Plasmodium falciparum* isolates can cause antifolate resistance and influence the course of antifolate resistance evolution. These findings showed the importance of *P. falciparum* GCH1 in drug resistance intervention. However, little is known about *P. falciparum* GCH1 in terms of kinetic parameters and functional assays, precluding the opportunity to obtain the key information on its catalytic reaction and to eventually develop this enzyme as a drug target.

**Methods:**

*Plasmodium falciparum* GCH1 was cloned and expressed in bacteria. Enzymatic activity was determined by the measurement of fluorescent converted neopterin with assay validation by using mutant and GTP analogue. The genetic complementation study was performed in *∆folE* bacteria to functionally identify the residues and domains of *P. falciparum* GCH1 required for its enzymatic activity. Plasmodial GCH1 sequences were aligned and structurally modeled to reveal conserved catalytic residues.

**Results:**

Kinetic parameters and optimal conditions for enzymatic reactions were determined by the fluorescence-based assay. The inhibitor test against *P. falciparum* GCH1 is now possible as indicated by the inhibitory effect by 8-oxo-GTP. Genetic complementation was proven to be a convenient method to study the function of *P. falciparum* GCH1. A series of domain truncations revealed that the conserved core domain of GCH1 is responsible for its enzymatic activity. Homology modelling fits *P. falciparum* GCH1 into the classic Tunnelling-fold structure with well-conserved catalytic residues at the active site.

**Conclusions:**

Functional assays for *P. falciparum* GCH1 based on enzymatic activity and genetic complementation were successfully developed. The assays in combination with a homology model characterized the enzymatic activity of *P. falciparum* GCH1 and the importance of its key amino acid residues. The potential to use the assay for inhibitor screening was validated by 8-oxo-GTP, a known GTP analogue inhibitor.

## Background

The folate pathway of *Plasmodium falciparum* is a well-established malaria drug target with proven benefits in treatment and prophylaxis [1,2]. The combination of antifolate pyrimethamine and sulphadoxine has been included in anti-malarial drug regimens for decades [3]. These antifolate compounds target two different enzymes in the folate pathway of *P. falciparum*, with pyrimethamine and sulphadoxine inhibiting dihydrofolate reductase (DHFR) and dihydropteroate synthase (DHPS), respectively [3]. The inhibition of the folate pathway cuts down the amount of folate derivatives that act as one-carbon carriers in nucleotide synthesis and amino acid metabolism. The malaria parasites became resistant to antifolates by gaining mutations at the *dhfr* and *dhps* genes [4-6]. The residue changes decrease the binding affinity of the drugs to the targeted enzymes [7,8]. The interest in antifolates has been renewed in recent years with the development of new lead compounds and the novel applications in malaria treatment [3,9]. Next-generation antifolates have now been developed in order to target drug-resistant folate enzymes [10]. The new P218 compound was designed to fit into the active site of pyrimethamine-resistant DHFR resulting in effective clearance of drug-resistant parasites [11]. Moreover, the existing antifolates can save the lives of infants and pregnant women at risk from malaria when administered as intermittent preventive regimens [9,12-14].

**Figure 1:**
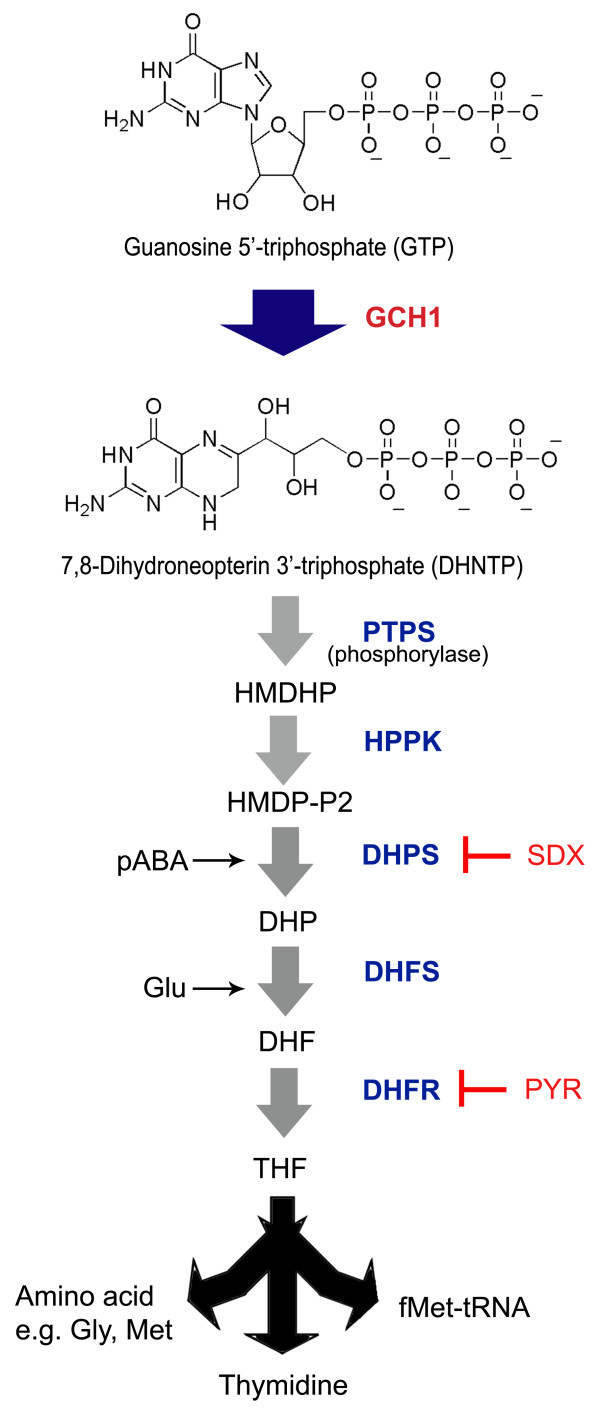
**GCH1 reaction in the folate pathway of *Plasmodium falciparum*.** Malaria parasites cannot salvage folate and need their own *de novo* folate pathway to synthesize folate derivatives [24]. GCH1 converts GTP to 7,8-dihydroneopterin 3′-triphosphate, which will become the pterin moiety of folate derivatives. Several *P. falciparum* strains were found to contain multiple copies of *gch1* (shown here as a large blue arrow). The next step in the folate pathway of *P. falciparum* is driven by 6-pyruvoyltetrahydropterin synthase (PTPS) to generate 6-hydroxymethyl-7,8 dihydroneopterin (HMDHP). It is worth noting that bacteria need an extra phosphorylase enzyme to remove the phosphate groups [25]. HMDHP is activated by the addition of two phosphate groups by hydroxymethyl dihydropteridine pyrophosphokinase (HPPK). Dihydropteroate synthase (DHPS) then combines the pterin moiety with 4-aminobenzoate (pABA) to produce 7,8-dihydropteroate (DHP). The last component to be added is glutamate via the reaction driven by dihydrofolate synthase (DHFS) to form 7,8-dihydrofolate (DHF). DHF is then reduced to 5,6,7,8-tetrahydrofolate (THF) by dihydrofolate reductase (DHFR). Anti-malarial sulphadoxine (SDX) and pyrimethamine (PYR) were combined to target two enzymes in the folate pathway of malaria parasites. For the chemical detail of the malarial folate pathway, see [2].

The rise in genomic analyses of malaria parasites revealed a unique role of GTP cyclohydrolase I (GCH1), the first and the rate-limiting enzyme of the folate pathway, in pyrimethamine resistance (Figure 1) [15]. Copy number polymorphism of *P. falciparum gch1* is found in malaria parasites from certain endemic countries, with some isolates from Thailand containing more than ten copies of *gch1*[16,17]. Extra *P. falciparum* GCH1 from gene amplification was shown to reduce pyrimethamine sensitivity slightly, but, most important of all, the extra enzyme could reduce the cost of drug-resistant mutations to the parasite during the gain of pyrimethamine resistance [18,19]. The increase in the rate-limiting GCH1 was found to improve the folate flux by several orders of magnitude [20]. The drug-resistant mutations at *dhfr*, though advantageous under pyrimethamine pressure, are costly in terms of fitness due to the changes at the active site [21-23]. Having extra rate-limiting GCH1 can boost the folate flux to compensate for the loss of the products in the pathway. The role of *P. falciparum* GCH1 in drug resistance evolution makes it necessary to characterize this enzyme biochemically and functionally. Understanding the properties of *P. falciparum* GCH1 will lead to the development of a new category of inhibitors that goes beyond killing an individual parasite. The inhibition of *P. falciparum* GCH1 might be able to prevent drug resistance evolution of other drug-targeted folate enzymes and could become a new strategy for fighting the emerging threat of malaria drug resistance.

GCH1 is a well-conserved protein found in bacteria, protozoa, plants and animals including human [26,27]. The enzyme converts GTP into 7,8 dihydroneopterin triphosphate, the precursor of the pterin moiety in folate derivatives (Figure 1) [26]. The enzyme forms a homodecameric barrel-like structure with ten zinc-containing active sites with each of them formed between three subunits [26]. The enzyme catalyzes the breakages of the guanine and ribose rings in GTP and rearranges them to form 7,8-dihydroneopterin triphosphate (Figure 1) [26,28]. The product was further processed by subsequent enzymes in the pathway, including DHPS and DHFR. In metazoa, GCH1 is controlled by GTP cyclohydrolase I feedback regulatory protein (GFRP) which acts as a negative regulator by binding to the N-terminus of GCH1 [29,30]. This N-terminal extension does not exist in the bacterial GCH1 proteins from *Escherichia coli* and *Thermus thermophilus*.

With a series of the new findings on the significance of *P. falciparum* GCH1, it is important to characterize this enzyme from malaria parasites. Here the biochemical properties of recombinant *P. falciparum* GCH1 were reported. The roles of the key residues and domains were tested by genetic complementation assays. A homology model was built to explore the overall structure and conserved residues at the active site. The information on the GCH1 enzyme could form a basis for the development of the chemical modulators of *P. falciparum* GCH1.

## Methods

### Plasmid construction

A series of *P. falciparum* GCH1 truncations was constructed by PCR cloning from pET45b(+)/GCH1 with *Pfu* DNA polymerase (Vivantis) and confirmed by direct sequencing [19]. The genomic DNA samples from the 7G8 and RO-33 *P. falciparum* strains (a gift from Dr Sarah Volkman, Harvard School of Public Health, Boston, MA, USA) were used to construct N88Y and R230K, respectively. H279S was made by QuikChange II (Agilent Technologies) with the pET45b(+)/GCH1 template. Each corresponding clone was inserted into pBAD33 with a ribosomal binding site.

### Functional complementation assay

Functional complementation was performed in *E. coli* K12 MG1655 *ΔfolE* (a gift from Professor Andrew Hanson, University of Florida, Gainseville, FL, USA). Construct was transformed by heat shock to *E. coli* K12 MG1655 *ΔfolE* with 300 μM thymidine supplement. Growth analysis was performed with preculture in LB broth (Bio Basic) supplemented with 300 μM thymidine (Sigma-Aldrich), 30 μg ml^-1^ kanamycin (Bio Basic) and 34 μg ml^-1^ chloramphenicol (Sigma-Aldrich). Starting culture was grown in the same media with 0.02% arabinose (Calbiochem) and without thymidine supplement at 37°C. Bacterial growth was determined using Spectrophotometer (Shimadzu UV-2501PC) at two-hour intervals. Each experiment was completed independently in at least triplicate*.*

### Protein expression and enzymatic assay

*Plasmodium falciparum* Δ1-195 GCH1 or core GCH1 was cloned into pET45b(+) and expressed in *E. coli* BL21(DE3)RIL with 0.4 mM isopropyl β-D-1-thiogalactopyranoside at 37°C for two hours. Protein was purified by Ni^2+^-sepharose (GE Healthcare) at 4°C in 50 mM NaH_2_PO_4_, 100 mM NaCl and 20% glycerol, pH 8 with 20 mM, 70 mM and 300 mM imidazole for binding, washing and elution, respectively. The purified protein was dialyzed against 50 mM Tris–HCl, 100 mM KCl and 20% glycerol, pH 7.8 at 4°C for 18 hours. The assay was performed according to a published protocol with minor modification [31]. In short, the complete reaction was composed of 50 mM Tris–HCl pH 7.8, 100 mM KCl, 20% glycerol, 250 μM GTP and 2.5 μM recombinant *P. falciparum* GCH1. The reaction was incubated in the dark at 37°C for 90 min and stopped with 67 mM HCl. Non-fluorescent 7,8-dihydroneopterin triphosphate was oxidized by 0.067% iodine (dissolved in 2% KI) to form fluorescent neopterin at room temperature in the dark for one hour. 0.12% ascorbic acid and 55.6 mM NaOH were then added. The product was measured by SpectraMax M5 (Molecular Devices) with neopterin standard (Sigma). All experiments for obtaining kinetic parameters were done in triplicate.

### Homology modelling

*Plasmodium falciparum* GCH1 was submitted to SWISS-MODEL homology modelling server [32]. A template model was *T. thermophilus* GCH1 (PDB: 1WUR) which served as a template for residue 203–383 of the conserved core domain [28]. The homology model for *P. falciparum* was obtained as a monomer. The decameric model was constructed in PyMol by superimposition onto *T. thermophilus* GCH1. The decameric model was further refined by optimizing side chain positions using Gromacs molecular dynamic package and GROMOS 43A1 force field [33]. The quality of the models was assessed by PROCHECK [34]. Secondary structure data were obtained from PDB accession number 1N3T, 1WUR, 1IS8 and 1FB1.

## Results

### Characteristics of *Plasmodium falciparum* GCH1

**Figure 2:**
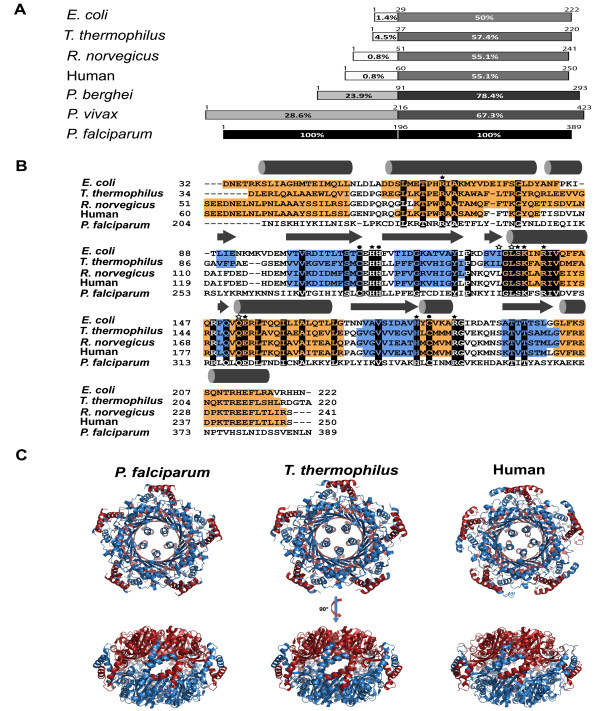
**Comparison of the GCH1 protein from *Plasmodium falciparum *to GCH1 proteins with known structures. (A)** Sequence alignment of the GCH1 proteins. The GCH1 sequences were divided into the N-terminal regulatory domain and the C-terminal enzymatic core with the residue numbers on each diagram. The homology scores compared to *P. falciparum* GCH1were shown as percent homology and colour shade (100% and black colour to its own sequence). **(B)** Secondary structure diagram from known GCH1 structures and the homology model of *P. falciparum* GCH1 with α-helices in orange and β-strands in blue. The secondary structure diagrams of the *P. falciparum* GCH1 model are at the top of the alignment. The conserved amino acid residues are highlighted in black with labelled key residues (see text for detail). **(C)** Comparison of the overall homodecameric GCH1 structures. Two face-to-face pentameric rings are coloured in red and blue.

*Plasmodium falciparum* PFL1155w (PF3D7_1224000) was shown to be malarial GTP cyclohydrolase I based on enzyme kinetics and complementation studies [19,35,36]. This is consistent with the GCH1 activity previously identified from parasite extract [37]. *Plasmodium falciparum* GCH1 was compared with the GCH1 sequences from the organisms with known protein structures [28,38,39]. The well-conserved core subunit of GCH1, which contains the active site, is located at the C-terminus of *P. falciparum* GCH1 (Figure 2A). The analysis of the N-terminal sequences showed a different picture with distinctively long unique sequences even among *Plasmodium* species (Figure 2A). The unique N-terminal sequence might suggest a regulatory mechanism unlike those GCH1 proteins from metazoan which are regulated by GFRP via the N-terminal domain. The homologue of GFRP has not been identified in *Plasmodium* species.

Comparative analysis between *P. falciparum* GCH1 and the GCH1 sequences from the species with structural data was performed. The key residues for coordinating with zinc are conserved with two cysteine and one histidine residues (black circles, Figure 2B) [39]. The fourth coordination with zinc was suggested to occur via a water molecule [28]. The residues that are shown by structural analysis to interact with GTP either via side chain (black stars, Figure 2B) or backbone (white stars, Figure 2B) are generally conserved. The homology model of *P. falciparum* GCH1 showed consistency in the overall structural component (Figure 2C). The N-terminus of *P. falciparum* GCH1 was excluded from the alignment and the model due to its uniqueness.

The homology model of *P. falciparum* GCH1 showed a similar overall structure at the core part. The core component of *P. falciparum* GCH1 was modelled and assembled into homodecamer based on previous structural determination [28]. The core component of GCH1 belongs to the T-fold protein family (T stands for tunnelling) [40]. Two pentameric rings are linked together by a clamp-like structure to form a face-to-face decameric barrel (Figure 2C). The tunnel in the middle of the decamer is formed by the last α-helix from every monomer. The active site is located on the external side of the barrel with ten of them formed between three subunits (the Homology model section for detail).

### Enzymatic properties of *Plasmodium falciparum* GCH1

In order to understand the biochemical properties of *P. falciparum* GCH1, its kinetic parameters were determined. The core domain of GCH1 (residue 196–389) from the 3D7 strain was chosen for this work because the core GCH1 protein still retains enzymatic and complementation activities. It is consistent with the fact that the core domain of GCH1 was found to assemble into a homodecameric structure with three subunits forming one active site. The core enzyme was found to be soluble and expressed well in bacteria compared to the full-length version probably from the lack of the long repetitive amino acid stretches at the N-terminus. The kinetic assays for *P. falciparum* GCH1 were performed, and fits of data gave K_m_ of 12.06 μM and k_cat_ of 0.039 s^-1^. The K_m_ values of GCH1 are in the micromolar range (4.2 μM and 31 μM for the GCH1 enzyme from *T. thermophilus* and human respectively) similar to the binding affinities of the SRP GTPase family [41]. It also means that the concentration of cellular GTP (~200-600 μM) exceeds the K_m_ value of GCH1 [42].

**Figure 3:**
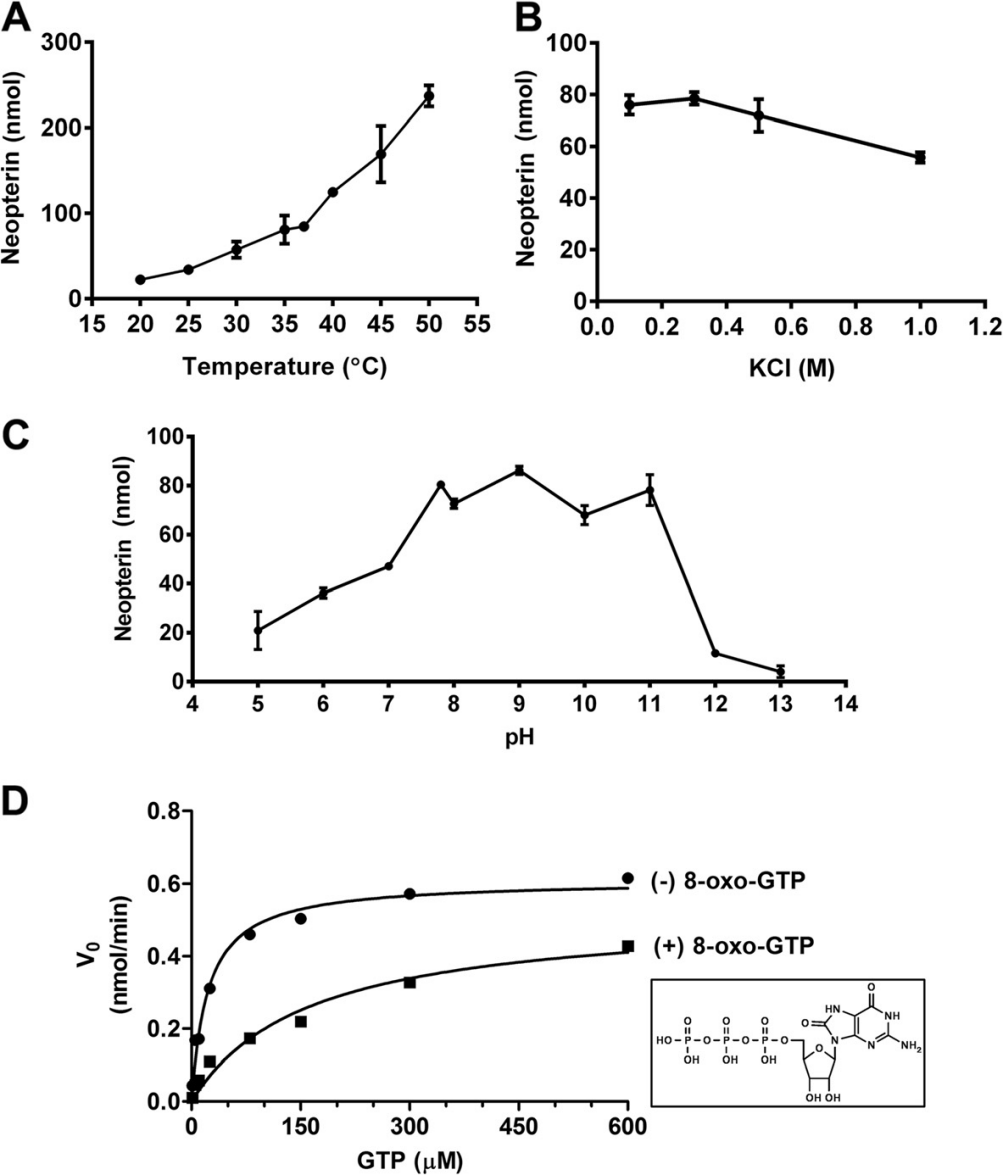
**Factors affecting the activities of *Plasmodium falciparum *GCH1. (A)** Effect of temperature shift on the GCH1 activity as shown by the production of the oxidized neopterin product. **(B)** Effect of salt (KCl) on the GCH1 activity. **(C)** Effect of pH change on the activity of *P. falciparum* GCH1. The pH values were varied from pH 5–13 with the data point from pH 7.8, which was chosen for the enzymatic assay. Every reaction in Figure 3A-3C was performed for 90 minutes. **(D)** Effect of 8-oxo-GTP on the activity of *P. falciparum* GCH1. The inhibitory effect on initial velocity was followed under various substrate concentrations.

The effects of temperature, salt and pH on the activity of *P. falciparum* GCH1 were then studied. The enzyme activity was found to be improved by the rise in temperature even at 50°C (Figure 3A). The finding indicates that feverish temperature in symptomatic malaria patients would not interfere with the activity of *P. falciparum* GCH1. High salt (1 M KCl) on the other hand could diminish the activity of the enzyme (Figure 3B). The best pH for the activity is at pH 9, but the activity peak is relative high over the broad pH range with a sudden drop when the pH reaching 12 (Figure 3C).

The enzymatic reaction of GCH1 was initiated by the attack of C8 of a guanine ring supposedly by a zinc-activated water. The modification of C8 would interfere with the enzymatic activity. 8-oxo-GTP was tested for its inhibitory effect on *P. falciparum* GCH1. As expected, 8-oxo-GTP could inhibit the activity of *P. falciparum* GCH1 with reduced fluorescent signal (Figure 3D). It shifted the V_max_ to 161.8 μM, but the value of k_m_ was not changed indicating that 8-oxo-GTP acts as a competitive inhibitor of GCH1. The overall enzymatic properties of GCH1 from the *P. falciparum* core domain and from other organisms are in the same range, consistent with the fact that the core GCH1 sequences are relatively conserved.

### Role of key residues and domains in GCH1 functional complementation

**Figure 4:**
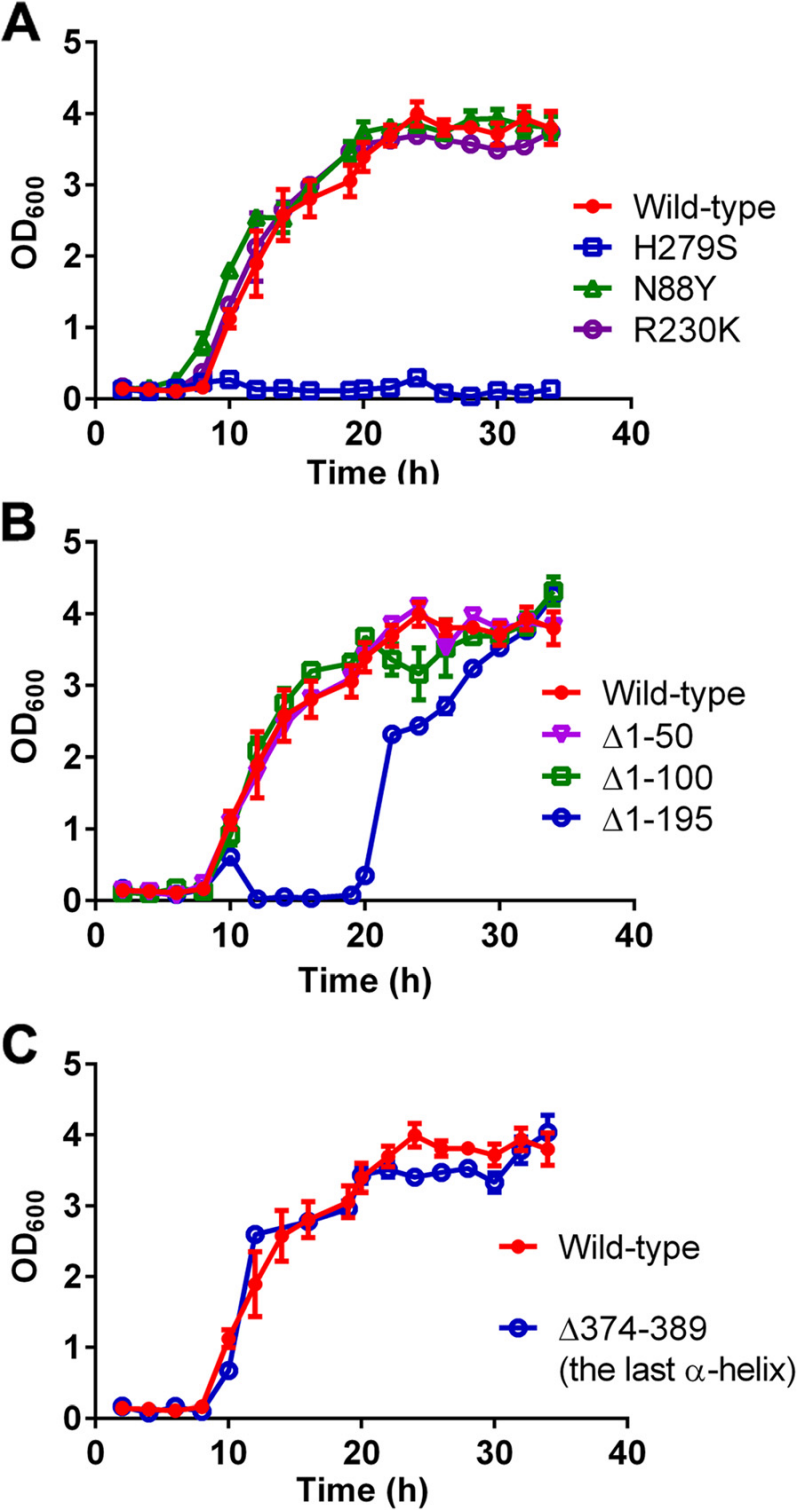
**Genetic complementation of *Plasmodium falciparum *GCH1 in bacteria. (A)** Mutation effect on *P. falciparum gch1* complementation. Wild-type *P. falciparum* GCH1 can rescue the loss of a bacterial strain without its own *gch1* (*folE* in bacteria). The loss of functional *P. falciparum* GCH1 as in the H279S mutant abolishes the complementation activity. Naturally-occurring mutations (N88Y and R230K) were also tested for their genetic complementation activities. **(B)** Effect of the N-terminal truncation on genetic complementation. A series of the N-terminal truncates was made in order to test their effect on genetic complementation. **(C)** Effect of the C-terminal helix deletion on genetic complementation. The well-conserved C-terminal helix was removed and tested for the complementation activity by the mutant.

A bacterial complementation assay was developed in order to identify the components of GCH1 that are necessary for its activity. The bacterial strains without *gch1 (∆folE*) was used as a model for genetic complementation [43]. *∆folE* requires thymidine supplement to survive. It was previously shown that the expression of *P. falciparum gch1* could rescue the loss of bacterial *gch1* allowing the growth in the condition without thymidine supplement [19]. The expression of wild-type *P. falciparum* GCH1 and mutant proteins in *∆folE* bacteria was induced by arabinose. Wild-type *P. falciparum* GCH1 can rescue the growth of *∆folE* bacteria in the condition without thymidine. Histidine 279 in *P. falciparum* GCH1 is a key residue for the enzymatic reaction (see below). The H279S mutant cannot rescue *∆folE* bacteria, indicating that the enzymatic activity of *P. falciparum* GCH1 is needed for genetic complementation (Figure 4A). The natural genetic diversity in *P. falciparum gch1* beside copy number polymorphism was also analyzed. Non-synonymous *gch1* mutations, N88Y and R230K, were identified and cloned from South American 7G8 and African RO-33 *P. falciparum* strains, respectively. The mutations were confirmed by direct sequencing and cloned into inducible vectors. Both mutants can still rescue the loss of bacterial *gch1* in *∆folE* bacteria (Figure 4A), which suggests that these naturally occurring mutations do not compromise basal enzymatic function.

The significance of the different domains in *P. falciparum* GCH1 was studied starting with the N-terminal domain. A series of the N-terminal truncates was made and tested for their complementation activities. The deletions of the first 100 amino acid residues do not cause any change in genetic complementation (Figure 4B). Even the removal of the entire N-terminal domain does not completely result in the loss of complementation like in the case of H279S, but the deletion of the entire N-terminal domain cannot reach the same level of complementation observed with that of the wild-type (Figure 4B).

Interestingly, two recent phosphoproteomic analyses independently identified protein phosphorylation at *P. falciparum* GCH1 especially at the N-terminus of *P. falciparum* GCH1 [44,45]. The control of GCH1 by protein phosphorylation was reported in *Drosophila melanogaster* as a positive regulator for GCH1 [46]. The mutagenesis of the phosphorylation sites in *D. melanogaster* GCH1 attenuated the enzyme function [46]. In *P. falciparum* GCH1, the phosphorylation sites were located at Ser109, Ser119 and non-canonical Cys117 [44]. The deletion of this part (∆1-195) slightly compromised GCH1 complementation, but no change was observed in the ∆1-50 and ∆1-100 truncates. This observation does not exclude the importance of the N-terminal domain on the function of *P. falciparum* GCH1, but it suggests that the N-terminal part might play a role in enzymatic control. The regulatory mechanism, perhaps via phosphorylation, is not likely to affect bacterial complementation assay used in this study. The unique N-terminal sequence of *P. falciparum* GCH1 compared to that of human indicates a different regulatory partner for the malarial enzyme. It could be an alternative target for developing plasmodial GCH1 inhibitors without a significant inhibitory effect on the human counterpart. The identification of the putative GCH1 kinase in *P. falciparum* could reveal the role of protein phosphorylation on the regulation of this enzyme.

The last helix of GCH1 that forms the lining of the tunnel was also investigated. The tunnel at the centre of the enzyme complex is common among the T-fold proteins. Interestingly, the tunnel in GCH1 contains additional α-helices from each monomer at the center of the tunnel (Figure 2C). This conserved feature was found in all GCH1 proteins from bacteria to metazoa. Surprisingly, the deletion of this helix does not affect genetic complementation at all (Figure 4C). This finding suggests that the last helix is not directly required for the catalytic activity of *P. falciparum* GCH1. Nevertheless, its high degree of conservation could imply the possibility of this helix to have another role such as in enzyme regulation and protein complex assembly.

### Homology model of *Plasmodium falciparum* GCH1

**Figure 5:**
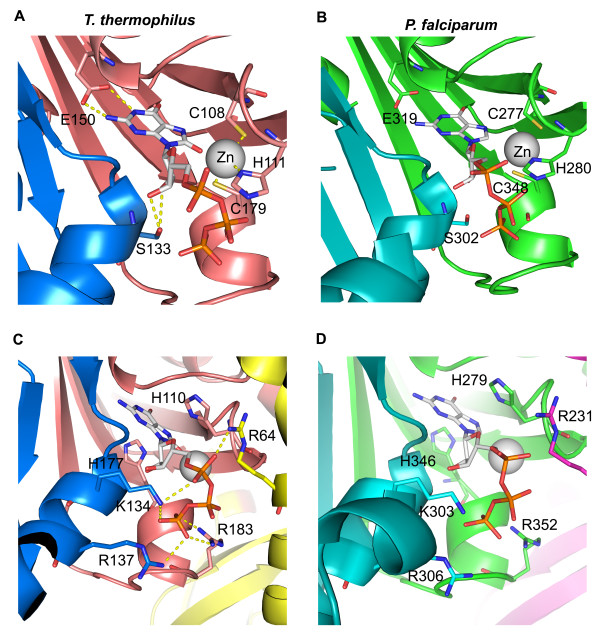
**Comparison of the *Plasmodium falciparum *GCH1 homology model and the GCH1 structure from *Thermus thermophilus*. (A)** and **(C)** the structure of *T. thermophilus* GCH1 showing the active site. **(B)** and **(D)** The homology model of *P. falciparum* GCH1 with the same views. See text for detail.

The homology model of *P. falciparum* GCH1 was built to observe the conserved residues with known functions based on *T. thermophilus* GCH1. The key residues for driving the enzymatic reactions and binding to the substrate are well conserved as expected. The active site of GCH1 is located at the interface between three subunits, two from the same pentameric ring and the other one from the opposite side. The X-ray structures of the GCH1 proteins from human, *E. coli* and *T. thermophilus* all suggested the presence of a metal ion bound to one histidine and two cysteine residues at each active site. The metal ion was found to be zinc by atomic absorption spectroscopy [39]. The zinc ion acts as Lewis base activating the water molecule to form hydroxyl nucleophile for nucleophilic attack at the guanine ring [38]. The residues in *T. thermophilus* GCH1 that coordinate with zinc are His 111, Cys 108 and Cys 179 (Figure 5A), which correspond to His 280, Cys 277 and Cys 348 in the *P. falciparum* GCH1 homology model (Figure 5B). *Thermus thermophilus* Glu 150 forms two hydrogen bonds with N1 and N2 of the guanine ring. This interaction could exist in *P. falciparum* GCH1 via conserved Glu 319. Another conserved chemical interaction is between the 2′ and 3′ hydroxyl groups of the ribose ring and Ser 133 (Ser 302 in *P. falciparum* GCH1) (Figure 5A and Figure 5B). Two His residues at the active site of *T. thermophilus* GCH1 were shown to participate in the catalytic reaction. *Thermus thermophilus* His177 (His 346 in *P. falciparum* GCH1) causes the protonation at N-7 in the guanine ring, which promotes the cleavage of N7/C8 at the guanine ring (Figures 5C and 5D) [28]. The replacement of the corresponding residue in *E. coli* GCH1 (His 179 in *E. coli*) results in the loss of enzymatic activity [47]. The second His residue is *T. thermophilus* His 110 (His 279 in *P. falciparum* GCH1) (Figures 5C and 5D). It acts as a hydrogen donor in the protonation of oxygen at the ribose moiety for the ribose ring breakage [28]. It might also participate in the C8/N9 imidazole ring cleavage to form formamidopyrimidine [28]. The triphosphate moiety of GTP is recognized by several basic residues located near the entrance of the active site pocket (Figure 5C). The structure of *T. thermophilus* GCH1 showed that Lys 134, Arg 137, Arg 183 and Arg 64 are responsible for the interactions with the phosphate groups. They correspond to Lys 303, Arg 306, Arg 352 and Arg 231 in the model of *P. falciparum* GCH1, respectively (Figures 5C and 5D).

The homology model revealed strong conservation of the key residues for substrate binding and conversion in *P. falciparum* indicating the strong selective pressure to maintain the enzymatic activity. The H279S mutant was constructed based on the homology model as a negative control and found to lose both the enzymatic and complementation activities. Human and *P. falciparum* GCH1 proteins are quite diverged especially at the residues lining the active site and substrate binding pocket. The experimentally solved structure of *P. falciparum* GCH1 is required to validate the observation based on homology modelling.

### Strategy for targeting *Plasmodium falciparum* GCH1

The enzymatic and complementation assays presented here have potential to be developed further for testing GCH1 inhibitors. A substrate analogue was tested to validate the capability of this assay to identify an inhibitor against plasmodial GCH1. *Plasmodium falciparum* GCH1 is an attractive drug target since it influences the course of drug resistance evolution [19], and it appears to be vital for erythrocytic-stage parasites as suggested by the failure to make a *gch1* knockout line in *P. falciparum*[1]. An inhibitor specific to *P. falciparum* GCH1 could be combined with antifolate inhibitors against DHFR and DHPS. The next-generation anti-folates such as P218 have already shown promising results in the assays with drug-resistant strains and liver-stage parasites [11,48]. The compounds that can effectively target liver-stage parasites with small side-effects are in high demand for prophylactic and relapse treatments. Nevertheless, cross-inhibition of putative plasmodial GCH1 inhibitors with human GCH1 needs to be avoided as well since human GCH1 is an essential enzyme in the production of tetrahydrobiopterin, a coenzyme in the production of key neurotransmitters and nitric oxide [49].

The inhibition of *P. falciparum* GCH1 has the potential to be a new strategy for drug resistance control especially with the new antifolate compounds currently under development [11,18,19]. Malaria drug resistance is a major obstacle to malaria elimination especially with the parasites from Southeast Asia, which are prone to develop drug resistance and contain highly diverged genetic repertoires [50,51]. Target inhibition of a factor contributing to drug resistance can be a novel strategy for overcoming malaria drug resistance.

## Competing interests

The authors declare that they have no competing interests.

## Authors’ contributions

KK, NK, PJ and TC carried out protein expression, purification and enzymatic assay. PC and KK performed complementation assay. PJ, TK and PC carried out plasmid constructions. NK and TK performed molecular modelling. YY and TC conceived of the study and coordination. NK, KK, PJA, YY and TC carried out data analysis. TC prepared manuscript. All authors read and approved the final manuscript.
